# Extracellular Vesicles from *Streptococcus suis* Promote Bacterial Pathogenicity by Disrupting Macrophage Metabolism

**DOI:** 10.3390/microorganisms13112469

**Published:** 2025-10-29

**Authors:** Wenjie Jin, Jinpeng Li, Zhaoyu Yi, Zhiheng Chang, Yue Li, Yamin Shen, Yingying Quan, Yuxin Wang, Baobao Liu, Li Yi, Yang Wang

**Affiliations:** 1 College of Animal Science and Technology, Henan University of Science and Technology, Luoyang 471000, China; 2 College of Life Science, Luoyang Normal University, Luoyang 471934, China; 3 Henan Provincial Engineering Research Center for Detection and Prevention and Control of Emerging Infectious Diseases in Livestock and Poultry, Luoyang 471003, China; 4 Animal Disease Prevention and Food Safety Key Laboratory of Sichuan Province, College of Life Sciences, Sichuan University, Chengdu 610065, China

**Keywords:** *Streptococcus suis*, extracellular vesicle, metabolism, pathogenesis

## Abstract

*Streptococcus suis* (*S. suis*) is an important zoonotic pathogen that causes severe disease in both humans and pigs, leading to substantial economic losses in the swine industry. Extracellular vesicles (EVs), as critical mediators of host–pathogen interactions, play key roles in bacterial virulence and disease progression. This study aimed to investigate the biological properties of *S. suis* EVs and elucidate their role in the bacterium’s pathogenesis. We isolated and characterized *S. suis* EVs, which were found to contain diverse protein molecules. EVs were efficiently internalized by mammalian cells, and concentrations below 50 μg/mL did not affect cell viability. Following uptake, EVs suppressed the production of key pro-inflammatory cytokines (*TNF-α*, *IL-1β*, and *IL-8*) by modulating macrophage metabolism. They also downregulated the expression of major histocompatibility complex class II (MHC-II) and adhesion molecules (*VCAM-1* and *ICAM-1*) during subsequent infections, potentially impairing macrophage-mediated clearance. In addition, EVs served as vectors for efficient cargo delivery and facilitated *S. suis* adhesion to and invasion of endothelial cells. In infection models, EVs markedly enhanced lethality in *Galleria mellonella* larvae and promoted tissue colonization in murine models. These findings suggest that *S. suis* EVs are key mediators of host–pathogen interactions, contributing to colonization and disease pathogenesis. Moreover, they offer novel insights and potential strategies for the prevention and control of *S. suis* infections.

## 1. Introduction

*Streptococcus suis* (*S. suis*) is a Gram-positive streptococcus that often colonizes the upper respiratory and digestive tracts of pigs. As an important zoonotic pathogen, it is capable of infecting humans via airborne transmission or contact with wounds [1,2,3], and in severe cases, it can cause a variety of infections including meningitis, bacteremia, endocarditis, and septicemia, and its pathogenicity is closely related to a variety of virulence factors and the immune status of the host. [2] There have even been outbreaks of large-scale *S. suis* infections of humans in Southeast Asia and some parts of China, and the World Health Organization has identified the *S. suis* infection as an important zoonosis affecting public health [4,5].

Extracellular vesicles (EVs) of Gram-positive bacteria have garnered significant attention in recent years. Unlike the outer membrane vesicles of Gram-negative bacteria, which lack an outer membrane structure, EVs are formed by protruding vesicles from the cytoplasmic membrane, which are secreted through the cell wall into the extracellular environment. Moreover, recent studies propose that bacterial explosive lysis is a new mechanism for EV production by Gram-positive bacteria [6]. These EVs are typically 40–400 nm in diameter and can carry a variety of bacterial components such as proteins, lipids, polysaccharides, and nucleic acids. There is growing evidence that some bacteria can utilize the release of extracellular vesicles as a mechanism for the delivery of virulence factors to the host cell. The role of EVs in virulence depends on their capacity to facilitate the transfer of bacterial components, including virulence factors, into eukaryotic cells [7]. This mechanism of intracellular delivery usually implies EV adhesion and internalization into the host cell. Extracellular vesicles, as a novel delivery vehicle, can enter the host cell in a variety of ways, including through cholesterol-enriched microdomain (lipid raft)-mediated endocytosis, receptor (lattice protein)-mediated endocytosis, and actin vesicle-mediated phagocytosis and cytosolic drinking [8], releasing its contents to exert functional effects. EVs’ internalization into the host cell is able to perform multiple functions, in addition to playing a direct role through the transport of toxins, proteases, and other substances, as well as through the membrane surface receptors to regulate the host immune response. Codemo et al. [9] reported that *Streptococcus pneumoniae* (*S. pneumoniae*) EVs carry agonists of TLR2 receptors such as teichoic acid and lipoproteins, which activate DCs and promote the production of inflammatory factors, and this phenomenon has been similarly reported in *Staphylococcus aureus* [10] and *Escherichia coli* [11]. Recent studies have also demonstrated that EVs not only play a role in acute infections but are also associated with chronic and recurrent infections. Internalization of *Brucella* OMVs into THP-1 cells inhibits the secretion of inflammatory factors and inhibits the formation of MHC-II molecules, hindering antigen delivery and promoting the survival of *Brucella* in macrophages [12]. Similar findings have been reported in *Porphyromonas gingivalis* (*P. gingivalis*), where *P. gingivalis* OMVs carry gingival proteases, leading to immune cell damage [13,14].

In this study, we demonstrated that EVs from *S. suis* with structurally intact membranes can deliver their contents into macrophages, modulate metabolic pathways, including glutamate metabolism, ammonia recycling, and the citric acid cycle, impede macrophage antigen delivery, inhibit *S. suis* clearance, and form persistent infections. At the same time, the EVs internalize the proteins they carry into the cells and promote *S. suis* adhesion and invasion of epithelial cells. Furthermore, results from the animal infection model were consistent with these observations, showing that exogenous supplementation with EVs enhances the pathogenicity of *S. suis*. The aforementioned phenomena may represent novel mechanisms underlying the establishment of persistent *S. suis* infections, thus providing fresh insights into addressing persistent *S. suis* infections.

## 2. Materials and Methods

### 2.1. Strain and Culture Conditions

*Streptococcus suis* (*S. suis*) HA9801 (hereafter referred to as HA) was used to isolate extracellular vesicles. *S. suis* virulent strain HA9801 was isolated in 1998 from Haian, Jiangsu Province; *S. suis* was cultured in trypticase soy broth (TSB) at 37 °C.

### 2.2. Isolation and Purification of EVs

Extracellular vesicles (EVs) were isolated from bacterial supernatants using a combination of ultra-high-speed centrifugation and size-exclusion chromatography (SEC) to reduce contamination and improve vesicle purity. The protocol described by Li et al. was followed with minor modifications [15]. Tryptic Soy Broth (TSB) medium was selected to support the optimal growth of *S. suis* was cultured at 37 °C with shaking at 180 rpm for 48 h. The resulting bacterial supernatant was then used for vesicle isolation. The bacterial culture was centrifuged at 4 °C at 12,000∗ *g* for 20 min to remove bacteria and insoluble particles. The collected supernatant was subsequently filtered through a 0.22 μm membrane. The supernatant was concentrated using a tangential-flow ultrafiltration system. The concentrated solution was ultracentrifuged at 100,000∗ *g* for 2 h at 4 °C. The supernatant was discarded, and the precipitate was resuspended in sterile PBS. This process yielded a crude EVs. The crude EV extract was then slowly loaded onto a size-exclusion column, with sterile PBS added to the column gradually. The 4th to 7th mL of effluent was collected and concentrated using a 10 kDa Millipore ultrafiltration tube to isolate the EVs for subsequent analysis. The EV samples were stored at 4 °C for short-term storage and at −80 °C for long-term storage.

### 2.3. Nanoparticle Tracking Analysis (NTA) to Measure Concentration and Particle Size of EVs

To determine the concentration and particle size distribution of the purified EVs, they were analyzed using ZetaView( Particle Metrix, Amsterdam, Bavaria, Germany). The PBS used for sample dilution was also tested prior to EV sample analysis to exclude any potential interference from PBS on subsequent experimental results. All EV samples were diluted in PBS to 1 × 10^5^–1 × 10^7^ particles/mL and subsequently introduced into the instrument’s sample chamber for nanoparticle tracking analysis to determine size distribution.

### 2.4. Determination of Protein Concentration in EVs

The concentration of protein carried by EVs was determined using the BCA Protein Assay Kit (Beijing Solarbio Science & Technology Co., Ltd, Beijing, China). A 5× RIPA lysis buffer was added to the isolated EVs, followed by mixing and incubation on ice for 30 min. Absorbance was measured at 562 nm using a microplate reader (Thermo Fisher Scientific, Waltham, MA, USA), and the samples were mixed prior to analysis. The protein concentration of the test samples was determined from a standard curve.

### 2.5. Scanning Electron Microscopy and Transmission Electron Microscopy Observations of EVs

The previously established methodology was followed with minor modifications [16], For SEM observation, the bacterial suspension was incubated at 37 °C, 180 rpm, for 48 h, then centrifuged at 4000 rmp for 10 min to collect the bacterial bodies, and then the cells were washed three times with PBS. The obtained cell precipitates were placed on cell coverslips to dry naturally and fixed with 2.5% glutaraldehyde for 4 h. The cells were subsequently dehydrated with ethanol through graded ethanol concentrations of 10%, 30%, 50%, 70%, 90%, and 100% for 20 min each. The cells were then dried, gold-coated, and observed by scanning electron microscopy (Zeiss, Oberkochen, Baden-Wurttemberg, Germany).

For TEM observation, purified *S. suis* EVs samples were adsorbed onto a 300-mesh copper grid, and then the grid was stained with 3% (*w*/*v*) phosphotungstic acid for 2 min, followed by two washes with PBS and one with distilled water. Finally, after adsorption on filter paper, the grid was air-dried for 10 min and observed under a transmission electron microscope (TEM, Hitachi, Tokyo, Japan) at 80 kV.

### 2.6. Cell Cultivation

Human laryngeal carcinoma epithelial cells (HEp-2) and mouse monocyte macrophage leukemia cells (RAW264.7) were used in this study. Both cell lines were cultured in DMEM medium supplemented with 10% heat-inactivated fetal bovine serum (FBS-303, Jin Yuan Kang Biotechnology, Inner Mongolia, China) at 37 °C in a 5% CO_2_ incubator.

### 2.7. Cytotoxicity Was Assessed by Calcein-AM/PI Staining of Cells

Following the method described by Wu et al. [17]. HEp-2 and RAW264.7 cells were cultured in 96-well plates according to the method described in cell cultivation until a confluent monolayer was formed. The cells were subsequently incubated with varying concentrations of EVs for 24 h. The cells were subsequently stained with Calcein-AM and propidium iodide (PI) for 30 min, followed by observation using a fluorescence microscope (Beckman Coulter, Shanghai, China).

### 2.8. Detection of EV Internalization by Flow Cytometry

According to the method described by Wang et al. [18] with minor modifications. EVs were labeled with FM4-64 and incubated with macrophages at varying concentrations in a 37 °C, 5% CO_2_ incubator for 4 h. To remove EVs adhering to the cell surface, cells were treated with a mixture of 0.5 mM sodium chloride and 0.5% glacial acetic acid for 45 s, followed by three washes with PBS and centrifugation. The processed cells were then analyzed using flow cytometry through the PE-Cy5-H channel, with the fluorescence intensity of the main cell population used as a measure of vesicle internalization. Data were processed and analyzed using the NovoExpress 4.0 software, with all experiments conducted in triplicate.

### 2.9. Fluorescence Microscopy of EV Internalization in Mammals

Based on the method described by Mehanny et al. [19], with slight modifications. HEp-2 and RAW264.7 cells were seeded into 96-well plates at a density of 5 × 10^3^ cells/well and incubated overnight at 37 °C in a 5% CO_2_ incubator until a monolayer was formed. FM4-64 (MCE, Wuhan, China)-labeled EVs were added to the culture medium and incubated for 4 h. To remove EVs adhering to the cell surface, cells were treated with a mixture of 0.5 mM sodium chloride and 0.5% glacial acetic acid for 45 s, followed by three washes with PBS. The processed cells were then fixed at room temperature with paraformaldehyde (Servicebio, Wuhan, China) for 15 min and washed three times with PBS. The cells were stained with 0.1 μg/mL DAPI (Thermo Fisher Scientific, Waltham, MA, USA) for 20 min and washed three times with PBS. The 96-well plates were mounted on the stage of an inverted fluorescence microscope (EVOS FL Auto, Thermo Fisher Scientific, USA) for observation.

### 2.10. Determination of the Effect of EVs on Cellular Activity

Based on the method described by Schulz et al. [20], with slight modifications. HEp-2 and RAW264.7 cells were cultured in 96-well plates according to the method described in cell cultivation until a monolayer was formed. The cells were then incubated with varying concentrations of EVs for 12 h, with PBS-treated cells (100% viability) serving as the negative control and 1% (*w*/*v*) DMSO-treated cells (no viability) as the positive control. A 10 μL aliquot of CCK-8 solution was added to each well, followed by incubation in the dark for 1 hour. Absorbance was then measured at 450 nm using a microplate reader.

### 2.11. Quantitative Reverse Transcription Polymerase

*S. suis* EVs purified at different concentrations (5 μg/mL, 25 μg/mL, 50 μg/mL) were added to cultured Raw264.7 cells (37 °C, 5% CO_2_) and incubated for 4 h. Then, *S. suis* (10^6^ CFU/mL) was added and placed in a 37 °C, 5% CO_2_ cell incubator for 4 h. Subsequently, RNA was extracted from *S. suis* using the TRIzol method. Total RNA from each group was reverse transcribed to obtain cDNA [21]. The transcript levels of target genes were quantified using quantitative reverse transcription polymerase chain reaction (RT-PCR) and the 2^-ΔΔCT^ method. All primer sequences used in this study are shown in Table 1.

### 2.12. Bacterial Adhesion and Invasion Assays

This assay was conducted based on the methodology described by Pan et al., with minor modifications [22]. The adhesion of *S. suis* to the human laryngeal carcinoma epithelial cell line (HEp-2) was evaluated. HEp-2 cells were cultured in 24-well plates according to the method described in cell cultivation until a confluent monolayer was established. Varying concentrations of EVs and 1 × 10^6^ *S. suis* were added to the cultured cells and incubated for 4 h. The cells were subsequently washed with PBS to remove non-adherent bacteria. Subsequently, 200 μL of 0.25% trypsin-EDTA was added to each well to lyse the cells for 5 min. The lysate was then diluted and plated onto tryptic soy agar (TSA) plates for bacterial enumeration. After incubation at 37 °C for 24 h, the colony-forming units (CFUs) were enumerated. The invasion assay followed procedures similar to those of the adhesion assay, with the key difference being the addition of 10^4^ μg/mL penicillin, 10^4^ μg/mL streptomycin, and 10^4^ μg/mL vancomycin to each well for 2 h after bacterial incubation with HEp-2 cells, to eliminate extracellularly adherent bacteria.

### 2.13. Different Treatments for EVs

The method described by Chorev et al. was followed with minor modifications [23]. For ultrasonic lysis-treated EVs (LEV), the purified EV was diluted with ammonium acetate, and the ultrasonic breaker pulse was set to 3 s on and 6 s off. The duration was 2 min and 30 s. The amplitude was set to 60% of the maximum value, and the ice-water bath was used during ultrasonic fragmentation for 20 min. The obtained protein and membrane mixture can be stored at 4 °C for 1–2 weeks. For heat-inactivated treated vesicles (HEV), boiling the purified EVs in a 100 °C water bath for 15 min is sufficient.

### 2.14. Metabolomics Analysis Based on LC-MS/MS

The effect of purified *S. suis* EV on the metabolome of Raw264.7 cells was evaluated based on the method of Husna et al. with minor modifications [24,25,26]. After culturing the cells, they were washed twice with pre-cooled PBS at 4 °C, and then the Petri dishes were placed on ice. One milliliter of pre-cooled methanol water (4:1, *v*/*v*) was added, and the cells were scraped from the sides of the dishes with a clean spatula. The cell solution was then aspirated into pre-cooled centrifugal tubes. The cells were centrifuged at 1000∗ *g* for 10 min, and the supernatants were discarded. The cells were then quenched with liquid nitrogen for 20 min. The cells were thawed at 4 °C, and 500 μL of extraction solution (acetonitrile: methanol: water = 2:2:1) was added. The cells were then broken by ultrasonication in an ice-water bath for 20 min. The cells were then centrifuged at 12,000 rpm for 10 min, and the supernatants were filtered through 0.22 μm filters. The resulting fractions were grouped for analysis. Each experimental group consisted of six biological replicates.

Off-target metabolite separation was performed using a Termo-Fisher UPLC system (Termo-Fisher, Waltham, MA, USA) coupled with an LTQ XL mass spectrometer (Thermo Fisher Scientific, Waltham, MA, USA). The mobile phase B consisted of methanol (HPLC grade, Merck, Germany), with a flow rate of 0.3 mL/min. A 2 μL of the sample was injected and the gradient process was as follows: 0–8 min (5% B), 8–18 min (35% B), 18–22 min (35% B), 22–28 min (90% B), 28–30 min (90% B), and 30–32 min (50% B). For mass spectrometry analysis, conditions were configured as follows: ESI ion source with a spray voltage of 3700 V (positive ion mode) or −3000 V (negative ion mode), a scanning range of 65 to 995 *m*/*z*, and a resolution of 70,000 at the first level and 17,500 at the second level. Collision energies were applied progressively at 3, 20 eV, 40 eV, and 60 eV. The scan rate was set to 7 Hz. After data acquisition, the resulting datasets were processed and visualized using MetaboAnalyst 6.0. [27]

### 2.15. Galleria mellonella (G. mellonella) Survival Experiments

Following the methodology outlined by Nadya V et al. with minor modifications [28]. *C* larvae were maintained in a 37 °C incubator, with 10 larvae per group. The larvae were cultured for at least 2 days prior to the experiment to allow for the restoration of their metabolic activity. A 10 μL aliquot of sterile PBS was injected into the final left proleg of each *G. mellonella* larva as the control group. A 10 μL injection of purified EVs at a concentration of 50 μg/mL was administered to the final left proleg of each *G. mellonella* larva as the EVs control group. In the experimental group, different concentrations of purified EVs (0 μg/mL, 5 μg/mL, 25 μg/mL, 50 μg/mL) were mixed with 5 × 10^5^ CFU/mL *S. suis* and 10 μL of the final mixture was injected into the final left proleg of each *G. mellonella* larva. The *Galleria mellonella* larvae were maintained at 37 °C for 3 days, with mortality recorded every 12 h. Survival data were analyzed using the Kaplan–Meier method, and intergroup differences were assessed for statistical significance with the log-rank (Mantel–Cox) test.

### 2.16. Pathogenicity Experiment in Mouse

Fifteen 4–6-week-old female Balb/c mice were randomly divided into three groups based on a previously established method [29]. One group served as the control, receiving no treatment; a second group was intraperitoneally injected with 200 μL *S. suis*; and the third group was intraperitoneally injected with 200 μL of a mixture containing purified *S. suis* EVs at a concentration of 50 μg/mL and 5 × 10^6^ CFU/mL. All mice were euthanized via intravenous injection of pentobarbital and subsequently dissected to obtain the liver, spleen, lungs, kidneys, and brain. The organs were placed in 1 mL PBS under sterile conditions, weighed, homogenized, serially diluted, and then plated onto TSA plates. The plates were incubated at 37 °C for 24 h, and colony-forming units (CFUs) were counted. The remaining organs were fixed in 4% paraformaldehyde (pH 7) for 36 h. Tissue samples were embedded in paraffin, sectioned at 4 μm thickness, and stained with hematoxylin and eosin. The samples were observed under an optical microscope (Weiyi Optoelectronics, Tianjin, China).

### 2.17. Statistical Analysis

All experiments were performed at least 3 times, and one-way analysis of variance (ANOVA), Student’s *t*-test, and two-way analysis of variance were performed using GraphPad Prism 9.5. For the *Galleria mellonella* survival assay, Kaplan–Meier survival curves were generated and intergroup differences were evaluated using the log-rank (Mantel–Cox) test. For CFU quantification, 10–100 was defined as a valid statistical interval and data not within the interval were discarded. Statistical analysis indicated a significant difference (ns: not significant, *, *p* < 0.05; **, *p* < 0.01; ***, *p* < 0.001).

## 3. Results

### 3.1. Isolation and Characterization of Extracellular Vesicles from Streptococcus suis (S. suis)

**Figure 1:**
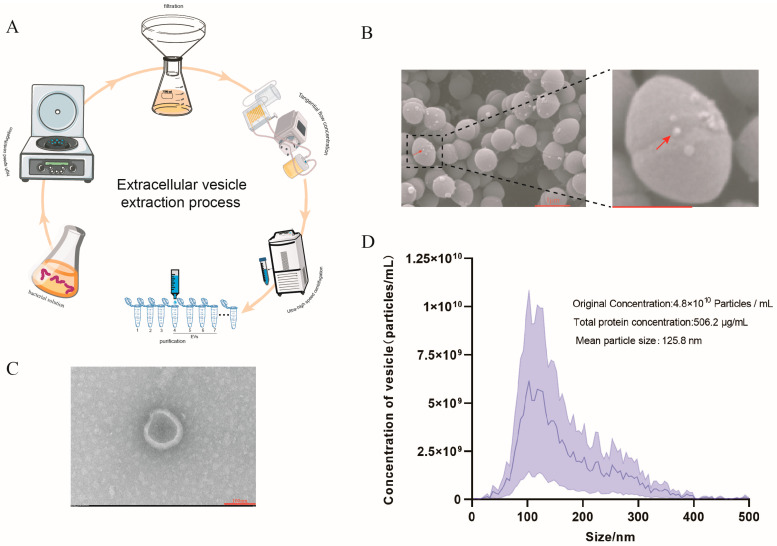
Isolation and Characterization of *S. suis* Extracellular Vesicles (EVs). (**A**) *S. suis* Extracellular Vesicle Isolation and Purification Flowchart. (**B**) Scanning electron microscopy (SEM) image of *S. suis*. The protrusions indicated by red arrows represent EVs The scale bar (red scale) in the magnified image represents 50 μm. (**C**) Transmission electron microscopy (TEM) image of isolated and purified *S. suis* EVs. (**D**) Nanoparticle tracking analysis of purified *S. suis* EVs, displaying the size distribution and particle concentration, as well as the average protein concentration carried by the vesicles.

To observe the production of extracellular vesicles (EVs) from *S. suis* HA9801, the morphology of *S. suis* was characterized using scanning electron microscopy (SEM). As shown in Figure 1B, a small number of EVs were observed on the surface of *S. suis* using SEM. The supernatant was concentrated using a 100 kDa tangential flow membrane, followed by ultracentrifugation to precipitate the EVs of *S. suis*. The isolated EVs were subsequently purified using a size exclusion chromatography column (Figure 1A). The purified EVs were then observed using transmission electron microscopy (TEM; Figure 1C) and nanoparticle tracking analysis (NTA; Figure 1D). The mean particle size of the isolated *S. suis* EVs was 125.8 nm. Additionally, the total number of particles obtained from the *S. suis* cultures at the final stage of the 500 mL platform was 2.4 × 10^12^, with an average particle concentration of 4.8 × 10^10^ particles/mL. The protein content of the purified EVs was determined to be 506.2 μg per milliliter using the BCA protein quantification kit (Beijing Solarbio Science & Technology Co., Ltd., Beijing, China).

### 3.2. S. suis EVs Are Internalized by Epithelial Cells (HEp-2) and Macrophages (RAW264.7) and Do Not Affect Cell Activity

**Figure 2:**
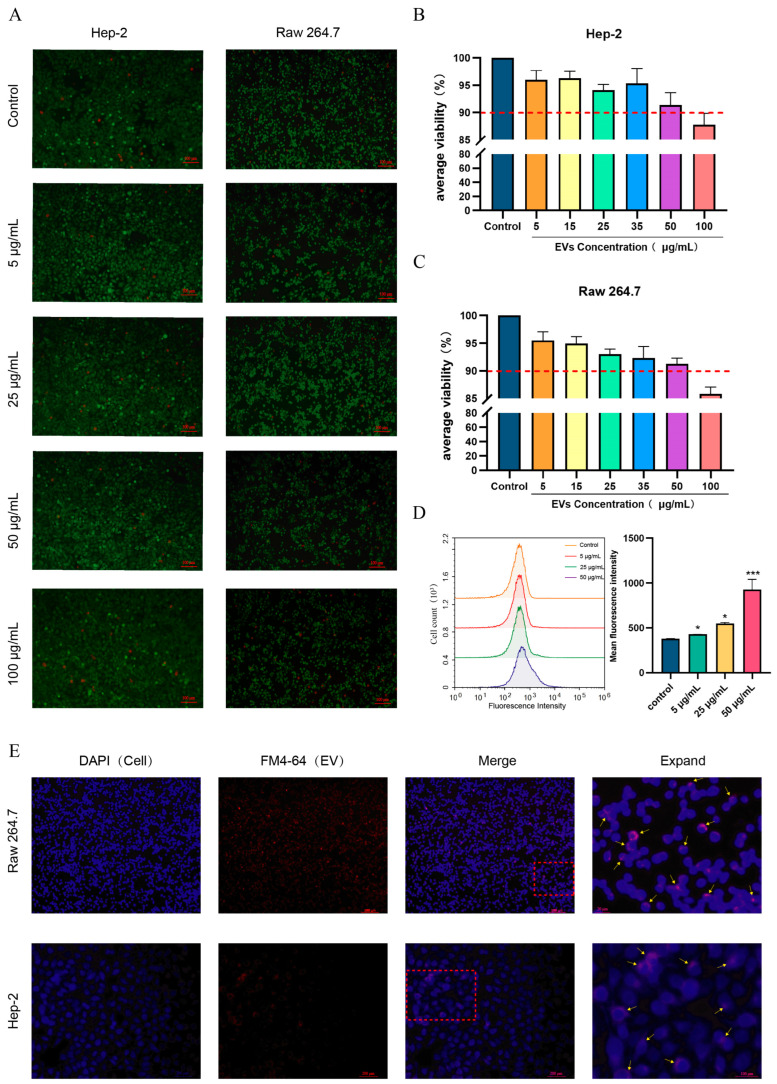
Internalization of *S. suis* EVs by Epithelial Cells (HEp-2) and Macrophages (RAW264.7) Without Affecting Cell Viability (**A**) Cell viability of HEp-2 and Raw264.7 cells, assessed by calcein-AM and PI staining after 12 h of preincubation with purified EVs. Green and red colors represent live and dead cells, respectively. (**B**,**C**) Percentage of cell survival, determined by CCK-8 assay, after preincubation with different concentrations of EVs. (**D**) Fluorescence intensity was detected by flow cytometry in Raw264.7 cells without EVs (control) and after 4 h of preincubation with various concentrations of FM4-64-labeled EVs. Bar graphs show the significance of the mean fluorescence intensity. Data are shown as the means ± SDs. * *p* < 0.05, *** *p* < 0.001 (**E**) Internalization of *S. suis* EVs into epithelial cells (HEp-2) and macrophages (Raw264.7) after 4 h of preincubation at 50 μg/mL EVs. Nuclei were stained with DAPI (blue), and EVs were labeled with FM4-64 (red). The area enclosed by the red dashed line is magnified on the right, and the yellow arrows indicate the sites where EVs are internalized into the cells.

We first assessed the cytotoxic effects of *S. suis* EVs on different cell lines. Mammalian epithelial cells (HEp-2) and macrophages (RAW264.7) were stained with calcein-AM and propidium iodide (PI) and subsequently observed using fluorescence microscopy. The results are presented in Figure 2A. Compared to the control group, no significant increase in cell death was observed following incubation with low concentrations of EVs (5–50 μg/mL). A noticeable increase in cell death was observed only after incubation with higher concentrations of EVs (100 μg/mL). We also detected cell activity after internalization using CCK-8, and the results were similar to the phenomenon observed by fluorescence microscopy. Significant impairment of cell activity was noted only after exposure to high concentrations of EVs (100 μg/mL) (Figure 2B,C). We next investigated whether *S. suis* EVs could be internalized by macrophages (RAW264.7). We selected a concentration of EVs that did not impair cellular activity, labeled them with FM4-64, and incubated them with the cells for 4 h. Flow cytometric analysis revealed a progressive shift in the fluorescence peaks to the right, accompanied by a significant increase in average fluorescence intensity as the EVs concentration increased (Figure 2D). Fluorescence microscopy was used to observe the internalization of EVs at the concentration exhibiting the most significant fluorescence variation (50 μg/mL). A weak red fluorescence signal was detected outside the cell nucleus, indicating that the labeled EVs were successfully internalized by macrophages. Similar observations were made in epithelial cells (HEp-2) at the same EV concentration (Figure 2E). These findings demonstrate that *Streptococcus suis*-derived EVs can be internalized by both murine macrophages and laryngeal epithelial cells. Furthermore, at relatively low concentrations, EVs did not exhibit a significant impact on cellular viability.

### 3.3. S. suis EVs Inhibit the Production of Cytosolic Pro-Inflammatory Factors, Reduce the Expression of MHC-II and Adhesion Molecules (ICAM-1 and VCAM-1), and Promote the Adhesive Invasion of S. suis

**Figure 3:**
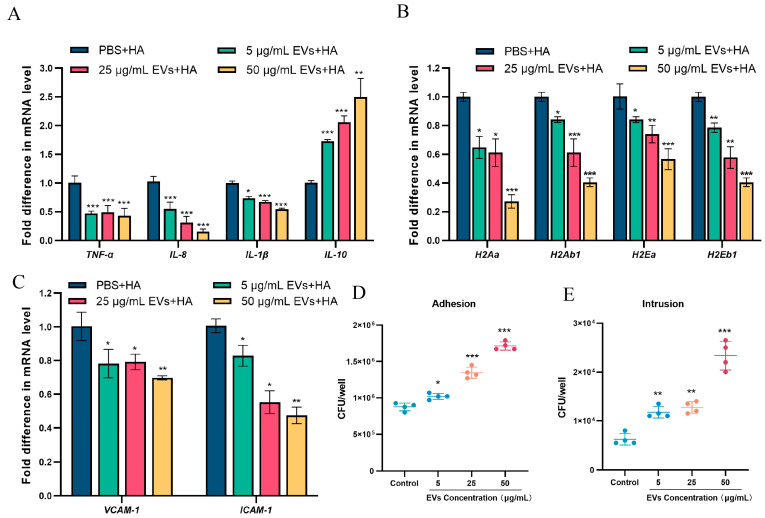
S. suis EVs inhibit the production of cytosolic pro-inflammatory factors and decrease the expression of histocompatibility antigen II (MHC-II) as well as cellular adhesion molecules (*ICAM-1* and *VCAM-1*), thereby promoting *S. suis* adhesion and invasion. (**A**) Raw264.7 cells were incubated for 4 h with different concentrations of EVs, followed by 4 h of *S. suis* infection with the addition of 10^6^ CFU/mL, and the mRNA transcription of major cytokines (*TNF-α, IL-8, IL-1β, IL-10*) was assessed. (**B**) The mRNA transcriptional levels of the *H2* gene cluster encoding MHC-II molecules. (**C**) The mRNA transcriptional levels of adhesion-related genes *VCAM-1* and *ICAM-1*. HA + PBS served as control group. (**D**,**E**) The number of *S. suis* adhering and invading to epithelial cells after 4 h of preincubation with various concentrations of EVs and 4 h of infection with the addition of 10^6^ CFU/mL *S. suis*. Data are shown as the means ± SDs. * *p* < 0.05, ** *p* < 0.01, *** *p* < 0.001.

Bacterial extracellular vesicles (EVs) are derived from the cytoplasmic or extracellular membrane and encapsulate membrane-associated proteins, virulence factors secreted by the bacteria, and components of the cell wall and extracellular membrane (e.g., phosphoglycolic acid, lipopolysaccharides). These vesicles have been shown to elicit an inflammatory response [6]. Consequently, we conducted experiments to evaluate whether porcine *S. suis* EVs could modulate the cytokine responses of murine macrophages during *S. suis* infection. Prior to *S. suis* infection, RAW264.7 cells were pre-incubated with purified EVs at varying concentrations to assess their effect on the transcriptional levels of inflammatory cytokines. The results are shown in Figure 3A. The transcriptional levels of pro-inflammatory factors (*TNF-α*, *IL-8*, *IL-1β*) was significantly reduced in cells pre-incubated with 50 μg/mL of EVs. Similar inhibitory effects were observed at lower concentrations of EVs (5 μg/mL, 25 μg/mL). Notably, in addition to inhibiting the transcription of pro-inflammatory factors, pre-incubation with *S. suis* EVs also enhanced the transcription of the anti-inflammatory cytokine *IL-10*, thereby contributing to a reduction in the host’s innate immune response. Therefore, we suspect that pre-incubation with porcine *S. suis* EVs suppresses the immune response of RAW264.7 cells and promotes immune evasion by *S. suis*. As crucial antigen-presenting cells, macrophages mediate specific immunity through phagocytosis, the processing of foreign pathogens, and the co-expression of histocompatibility antigen II on the cell membrane, which is essential for presenting antigens to helper T cells to clear pathogenic bacteria [30]. Accordingly, we assessed the transcriptional levels of *H2*-related genes encoding MHC-II molecules in RAW264.7 cells following pre-incubation with varying concentrations of EVs for 4 h before *S. suis* infection. In the absence of *S. suis* infection, treatment of RAW264.7 cells with 50 μg/mL EVs alone did not cause a significant change in the transcriptional level of the H2-related gene compared with the PBS-treated group (Supplementary Figure S1). However, when RAW264.7 cells pretreated with 50 μg/mL EVs were subsequently infected with *S. suis*, the transcriptional level of the H2-related gene was markedly reduced compared with cells subjected to infection alone, and a similar inhibitory effect was observed at lower EV concentrations (5 μg/mL and 25 μg/mL) (Figure 3B). When macrophages interact with helper T cells and present antigens via MHC-II molecules, they also upregulate the expression of intercellular adhesion molecule (*ICAM-1*) and vascular cell adhesion molecule (*VCAM-1*) to enhance adhesion to helper T cells and facilitate antigen delivery. These experiments showed that EVs could inhibit the transcription of *H2*-related genes encoding MHC-II molecules. Thus, we next investigated whether EVs also affect the transcription of macrophage adhesion-related genes. To this end, we quantified the gene transcriptional levels of *ICAM-1* and *VCAM-1* during *S. suis* infection after 4 h of preincubation with different concentrations of EVs. In the non-infection group, no significant difference in *ICAM-1* expression was observed between the PBS-treated cells and those treated with 50 μg/mL EVs (Supplementary Figure S1). However, the transcription of *VCAM-1* was undetectable in both PBS- and EV-treated cells, which is likely attributable to the fact that *VCAM-1* expression occurs predominantly in activated macrophages. In the *S. suis*-infected group, pre-incubation with EVs at varying concentrations significantly inhibited the transcription of *ICAM-1* and *VCAM-1*. Notably, in cells pre-incubated with 50 μg/mL of EVs, the transcriptional level of *ICAM-1* was reduced to half of that observed in the *S. suis*-infection-only group. These results suggest that *S. suis* EVs can down-regulate the expression of MHC-II and cell adhesion molecules during *S. suis* infection, thereby inhibiting antigen presentation during the infection process, which may facilitate *S. suis* colonization and invasion in the early stages of infection. Therefore, we wondered whether EVs play a role in *S. suis* adhesion and intrusion. To verify this possibility, we added different concentrations of purified EVs to *S. suis* during the infection of human epithelial cells (HEp-2) to determine the number of adherent and invading bacteria. The results are shown in Figure 3D,E. Following the exogenous addition of EVs (50 μg/mL, 5 μg/mL, and 25 μg/mL), the number of S. suis adhering to and invading HEp-2 cells was significantly increased, with a positive correlation observed with EVs concentration.

### 3.4. Only EVs with Intact Membrane Structures Function as Carriers to Deliver Contents into Cells

**Figure 4:**
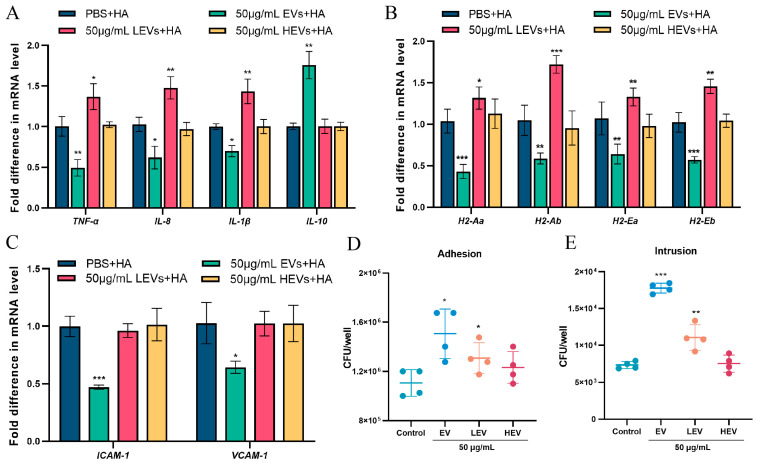
EVs with an intact membrane structure function as carriers, delivering contents into cells. (**A**) The mRNA transcription of major cytokines (*TNF-α, IL-8, IL-1β, IL-10*) in Raw264.7 cells incubated with 50 μg/mL of differently treated EVs for 4 h, followed by addition of 10^6^ CFU/mL of *S. suis* for 4 h of infection. (**B**) The mRNA transcriptional levels of the *H2* gene cluster encoding MHC-II molecules. (**C**) The mRNA transcriptional levels of adhesion-related genes *VCAM-1* and *ICAM-1*. HA + PBS served as control group. (**D**,**E**) The number of *S. suis* adhering and invading to epithelial cells after 4 h of preincubation with 50 μg/mL of differently treated EVs, followed by 4 h of *S. suis* infection with the addition of 10^6^ CFU/mL. Data are shown as the means ± SDs. * *p* < 0.05, ** *p* < 0.01, *** *p* < 0.001.

The aforementioned results suggest that *S. suis* EVs can inhibit antigen delivery and promote *S. suis* colonization and immune evasion. Thus, we hypothesize that the intact vesicle membrane structure plays a crucial role in this process. To verify this hypothesis, we subjected purified *S. suis* extracellular vesicles to ultrasonic lysis (which destroys vesicle structure while retaining the intact vesicle contents, LEV) and high-temperature boiling inactivation (which destroys the vesicle structure while heat-inactivating vesicle-associated proteins, HEV). After pre-incubation of *S. suis* with mouse macrophages for 4 h prior to *S. suis* infection, the transcriptional changes in key cytokines, MHC-II molecules, and cell adhesion-related genes were assessed. The results are presented in Figure 4A. The transcriptional levels of inflammatory factors in macrophages pre-incubated with LEV and infected with *S. suis* increased significantly, exhibiting a clear opposite trend compared to the EV-treated group, The HEV-treated group showed no significant changes. The transcriptional changes in *H2*-related genes were similar to those observed in cytokines, with LEV pre-incubation promoting the transcription of the *H2* gene cluster, whereas HEV pre-incubation showed no significant effect (Figure 4B). In contrast, the transcriptional changes in *ICAM-1* and *VCAM-1* differed from the patterns described above. Compared with the *S. suis*-infection-only group, EV treatment significantly reduced their transcription, whereas neither LEV nor HEV pre-incubation resulted in notable changes (Figure 4C). We also investigated the effects of different vesicle treatments on the adhesion and invasion of *S. suis* to epithelial cells. The results are presented in Figure 4D,E. HEV treatment had no effect on the adhesion and invasion of *S. suis*. However, pre-incubation with EVs and LEVs significantly promoted the adhesion and invasion of *S. suis* to epithelial cells, with EV co-incubation demonstrating a more pronounced effect. This phenomenon may be due to the fact that EVs carry *S. suis* colonization-associated proteins that do not need to enter the host cell to function. These results indicate that only *S. suis* EVs with an intact membrane structure can facilitate bacterial immune evasion. While vesicles lacking a complete membrane structure also promote *S. suis* colonization, their effectiveness is inferior to that of intact EVs.

### 3.5. The Internalization of S. suis EVs Induces Metabolic Changes in Macrophages

**Figure 5:**
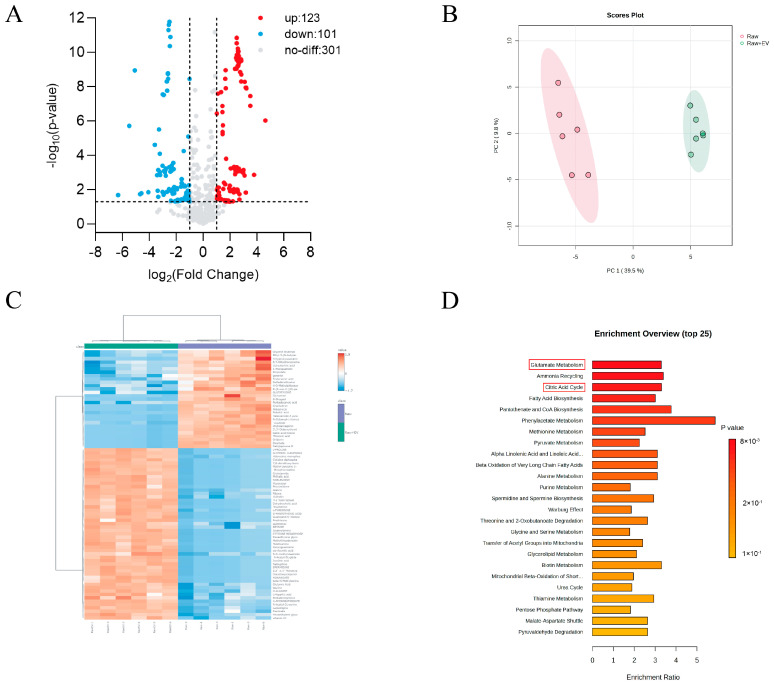
The internalization of *S. suis* EVs induces metabolic changes in macrophages. (**A**) Volcano plot of differential metabolites detected between Raw264.7 cells and Raw264.7 cells incubated with 50 μg/mL of EVs. (**B**) Principal component analysis (PCA) of differential metabolites between RAW264.7 cells and Raw264.7 cells incubated with EVs. (**C**) Heatmap analysis of differential metabolites between Raw264.7 cells and Raw264.7 cells incubated with EVs (**D**) KEGG pathway analysis of differential metabolites between Raw264.7 cells and Raw264.7 cells incubated with EVs, where the red box highlights the differential metabolite enrichment pathway associated with the red box highlighting. Data are shown as the means ± SDs.

We hypothesize that the observed changes in cytokines and MHC-II molecules may be attributed to alterations in macrophage metabolism. To investigate this, we performed LC-MS/MS analysis to compare the metabolic differences in RAW264.7 cells pre-incubated with 50 μg/mL EVs for 4 h. As shown in Figure 5A, EV-preincubated Raw264.7 cells exhibited 224 differential metabolites, of which 123 were upregulated and 101 were downregulated, compared to Raw264.7 cells alone. And principal component analysis (Figure 5B PCA) and clustered heatmap analysis (Figure 5C) demonstrated that the levels of a variety of immune-related metabolites appeared to be significantly altered after *S. suis* EVs preincubation, emphasizing that the significant effect of EVs on macrophage metabolism. To further elucidate the functions of these differential metabolites, we performed a KEGG pathway analysis. The results are shown in Figure 5D. These differential metabolites were mainly focused on glutamate metabolism, ammonia cycle, citric acid cycle, and fatty acid biosynthesis. Recent studies have shown that glutamine plays an important role in macrophage activation as well as proliferation [31]. Glutamine is involved in both glutamate metabolism and ammonia cycling, and our data show a significant reduction in glutamine content in macrophages following EVs incubation. Additionally, we observed a significant increase in isocitrate and cis-aconitate, with isocitrate formed through the conversion of citric acid. Citrate, in turn, has been shown in several studies to exhibit pro-inflammatory activity and play an important role in host defense against pathogenic bacteria. In activated dendritic cells (DCs), the isocitrate formation pathway within the TCA cycle is blocked, leading to citrate accumulation. [32]. In summary, these findings clearly demonstrate that the internalization of *S. suis* EVs alters the metabolism of murine macrophages, playing a crucial role in bacterial immune evasion.

### 3.6. Animal Model to Assess the Effect of Extracellular Vesicles on the Pathogenicity of S. suis

**Figure 6:**
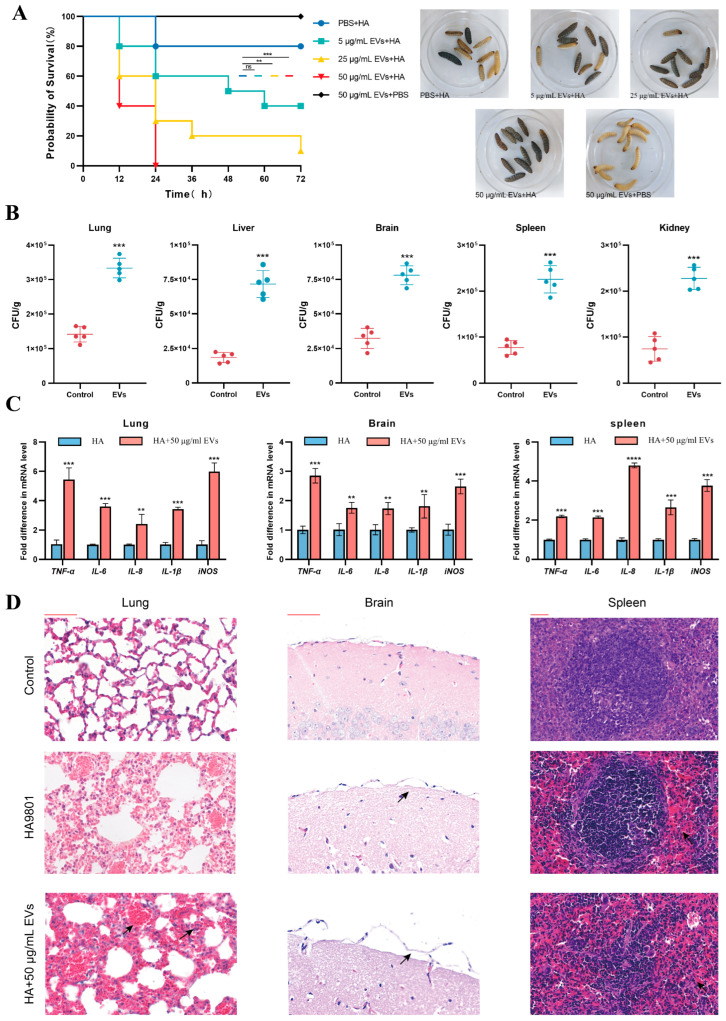
Animal models to assess the effect of extracellular vesicles on the pathogenicity of *S. suis* (**A**) Lethality of *G. mellonella* following mixed infection with various concentrations of EVs and *S. suis*. (**B**) Bacterial load in various organs of mice subjected to mixed treatment with 50 μg/mL EVs and *S. suis*. (**C**) The mRNA transcription of major inflammatory factors (*TNF-α, IL-6, IL-8, IL-1β, iNOS*) in the lungs, brains and spleens under mixed treatment of 50 μg/mL. (**D**) Pathological changes in the lungs, brains, and spleens of mice following mixed treatment with 50 μg/mL EVs and *S. suis.* The black arrows indicate areas with exacerbated pathological changes. Scale bar (red scale): 50 μm. Data are shown as the means ± SDs. ** *p* < 0.01, *** *p* < 0.001, **** *p* < 0.0001. ns *p* > 0.05.

The previous results demonstrated that EVs with intact vesicle membrane structures can internalize into cells, alter macrophage metabolism, inhibit antigen presentation, promote immune evasion by *S. suis*, and play a critical role in its colonization and infection. Thus, we aimed to assess whether EVs exert a similar effect during *S. suis* animal infections. The effect of EVs on the pathogenicity of *S. suis* was evaluated using a *G. mellonella* infection model. As shown in Figure 6A, infection of *G. mellonella* with 50 μg/mL of EVs alone resulted in 100% survival after 72 h, indicating that *S. suis* extracellular vesicles are not harmful to *G. mellonella*. Infection of *G. mellonella* with HA alone resulted in 80% survival after 72 h, which may be attributed to the relatively low pathogenicity of *S. suis* at this bacterial load. However, the addition of *S. suis* extracellular vesicles exogenously resulted in varying degrees of reduction in the survival rate; The survival rate of *G. mellonella* was reduced to 40% and 10% after 72 h with the exogenous supplementation of lower concentrations of EVs (5 μg/mL and 25 μg/mL), respectively. These findings suggest that the exogenous addition of *S. suis* EVs significantly reduces the survival rate of *G. mellonella* and enhances the pathogenicity of *S. suis*, with the enhancement of pathogenicity positively correlated with the concentration of supplemented EVs. We then selected the concentration of EVs that exhibited the most potent immunosuppressive effect in cellular experiments and utilized a mouse model to assess the ability of EVs to mediate immune evasion by *S. suis* in vivo. As shown in Figure 6B, the exogenous addition of 50 μg/mL of *S. suis* EVs significantly increased bacterial load in various tissues and organs of mice compared to *S. suis* infection alone. Next, we selected the most suitable organs for *S. suis* colonization (lungs and brain) and the spleen, where the change in bacterial load was more significant, for the measurement of inflammatory factors and the preparation of organ pathology sections. As shown in Figure 6C, the infection group supplemented with EVs exhibited different increases in major inflammatory factors compared to the *S. suis* infection group alone.; Pathological sections (Figure 6D) further revealed that, compared to the organ damage in the HA-infected mouse group, the group supplemented with exogenous *S. suis* EVs exhibited signs of increased damage. This was characterized by significant congestion in brain tissue, more severe detachment of the meninges, increased hemorrhage in the spleen, and congestion of lung alveolar cavities with inflammatory cell infiltration. These results suggest that *S. suis* EVs mediate immune evasion in mice and enhance the pathogenicity of *S. suis*.

## 4. Discussion

*Streptococcus suis* (*S. suis*), a zoonotic pathogen, frequently causes persistent infections, poses a significant safety threat to humans, and imposes a substantial economic burden on the global pig industry [33]. Previous studies have demonstrated that *S. suis* can establish chronic infections in the host through multiple immune evasions mechanisms, such as the production of enolase, factor H-binding proteins, and a two-component regulatory system. These factors contribute to immune evasion by preventing host phagocytosis and disrupting complement-mediated innate immunity, leading to diseases such as meningitis and arthritis [34]. Several recent studies have shown that extracellular vesicles produced by bacteria, in some cases, also have immunomodulatory effects that favor the establishment of chronic infections. For example, extracellular vesicles of *S. pneumoniae* can recruit factor H, inactivate complement protein C3, and disrupt complement-mediated immunity, thereby promoting immune evasion by the bacteria [9]. Outer membrane vesicles from *Brucella abortus* promotes the establishment of persistent infections by inhibiting the production of proinflammatory factors and the expression of MHC-II molecules, thereby hindering antigen presentation [12].

In the present study, we investigated the interaction of extracellular vesicles (EVs) with phagocytes and epithelial cells and assessed the impact of this interaction on host cellular immunity during *S. suis* infection. The EVs isolated and purified from *S. suis* in this study were similar in size to those described in previous studies (*S. pneumoniae* EVs range from 25–250 nm, [19], and *S. suis* type 2 EVs range from 150–300 nm [35]). The diameters of *S. suis* EVs in our study ranged from 30–400 nm, and this variation may be attributed to differences in bacterial culture conditions, vesicle isolation methods, and purification techniques. The observed difference in EVs size compared to the literature prompted us to further investigate *S. suis* vesicles using electron microscopy. SEM imaging revealed small vesicles protruding from the bacterial cell wall, which may indicate the ongoing shedding of *S. suis* EVs. In the next experiments, we explored the effects of EVs of *S. suis* on epithelial cells and macrophages. Using the CCK-8 assay and fluorescence microscopy, we found that only higher concentrations of EVs (100 μg/mL) affected the growth of mammalian cells (HEp-2 and RAW264.7), whereas lower concentrations had no observable effect. This phenomenon is similar to the findings reported by Mehanny et al. [19], who demonstrated that *S. pneumoniae* EVs were not only non-toxic to A549 cells but can even promote their growth. Subsequently, flow cytometry and fluorescence microscopy revealed the successful internalization of *S. suis* EVs into various mammalian cells after 4 h of pre-incubation. This phenomenon of vesicle internalization into host cells has also been reported for other bacteria, such as *S. pneumoniae* and *Brucella abortus*. These results suggest that EVs can serve as carriers for *S. suis* antigens, delivering them into host cells. This novel antigen delivery system enables antigens to interact with host cells prior to direct contact with *S. suis*, thereby influencing subsequent bacterial infection. This interaction likely underlies the experimental observations made in this study.

Bacterial extracellular vesicles (EVs) carry not only various virulence proteins but also innate immune activators, such as lipopolysaccharides and phosphoglycolic acid [6], EVs can induce either enhanced or attenuated immune responses upon interacting with the host. Previous studies have shown that EVs stimulates the secretion of cellular inflammatory factors (*TNF-α, IL-8, IL-1*, etc.), such as those reported for *Escherichia coli* [11], *Staphylococcus aureus*, *Bacteroides fragilis* [10] and many other bacteria. In contrast, some bacterial EVs can downregulate the immune response and inhibit the production of inflammatory factors. For example, outer membrane vesicles (OMVs) from *Brucella abortus* can inhibit the production of inflammatory factors in THP-1 cells [12]. Vesicles from *Akkermansia muciniphila* reduce *IL-6* production and increase *IL-10* secretion, thereby alleviating carbon tetrachloride-induced pulmonary fibrosis in mice [36]. *S. suis* EVs serve as novel carrier systems, capable of delivering a wide range of bacterial antigens, including virulence factors, to host cells. In this study, we further evaluated the effect of EVs on the production of inflammatory factors in macrophages. We observed that pre-incubation with *S. suis* EVs significantly inhibited the production of inflammatory factors (*TNF-α, IL-1β, IL-8*) in RAW264.7 cells during *S. suis* infection. *TNF-α*, an endogenous cytokine that orchestrates gene expression and cellular activity and generates inflammatory signals to recruit other immune cells to trigger an immunological cascade response, plays an important role in the regulation of immune cells [37,38]. We suggest that inhibition of *TNF-α* expression attenuates cellular inflammation and suppresses the subsequent immune response. Notably, RAW264.7 cells pre-incubated with *S. suis* EVs produced significantly higher levels of *IL-10* during subsequent infections. *IL-10* exhibits broad anti-inflammatory activity, primarily targeting macrophages and dendritic cells (DCs). It inhibits antigen presentation, reduces the expression of major histocompatibility complex class II (MHC-II) molecules, hinders antigen-presenting cell (APC) maturation, and decreases the secretion of proinflammatory cytokines by immune cells [39]. The above results suggest that EVs can affect cytokine production and may inhibit antigen delivery. This is further supported by our finding that EVs pre-incubation significantly decreases the expression of MHC-II molecules. MHC-II molecules on the surface of immune cells process antigens from pathogenic bacteria into peptide fragments and present them to helper T cells. Helper T cells recognize the antigen, becoming activated and subsequently inducing an immune response [30]. Therefore, EV-mediated downregulation of macrophage MHC-II expression attenuates the host immune response and promotes immune evasion by *S. suis*. In addition to MHC-II molecules, macrophages also express intercellular adhesion molecules (*ICAM-1*) and vascular cell adhesion molecules (*VCAM-1*) on their surface. These molecules play a crucial role in innate immunity by promoting macrophage translocation to infection foci and facilitating the immobilization of helper T cells to assist in antigen delivery [40]. Our study found that EVs pretreatment significantly inhibited and dose-dependently downregulated the expression of *ICAM-1* and VCAM-1 on macrophages during *S. suis* infection. Pollak et al. [12] previously reported that OMVs from *Brucella abortus* downregulate the expression of intercellular adhesion molecules *ICAM-1* and *VCAM-1*. These observations support our hypothesis that the interaction between *S. suis* EVs and mouse macrophages inhibits the production of inflammatory factors during subsequent *S. suis* infection. Additionally, it suppresses the expression of MHC-II molecules and adhesion molecules (*ICAM-1* and *VCAM-1*), which impairs antigen delivery, hinders the immune system’s clearance of *S. suis*, and facilitates the establishment of a persistent infection. This EV-host cell interaction may represent a novel mechanism of immune evasion by *S. suis*.

EVs from *Actinobacillus actinomycetemcomitans* were reported to enhance their adhesion to oral epithelial cells [41]. EVs of *S. pneumoniae* promote their adhesion and invasion to human monocyte macrophages [19]. Similar to these findings, we observed in this study that the exogenous addition of EVs during bacterial infection significantly enhanced *S. suis* adhesion to and invasion of human laryngeal epithelial cells (HEp-2) in a dose-dependent manner. These results suggest that EVs released by *S. suis* during interactions with HEp-2 cells enhance bacterial colonization and invasion of epithelial cells, aiding *S. suis* in establishing persistent infections in the host. We hypothesize that this phenomenon may be linked to the enolase carried by *S. suis* EVs. We speculate that this phenomenon may be related to bacterial adhesion proteins carried by *S. suis* EVs, such as enolase. For example, multiple studies have shown that EVs from *S. suis* serotype 2 (P1/7, Ss2) contain enolase (Eno) [42]. Eno not only facilitates *S. suis* adhesion to host cells but also aids in bacterial penetration of the blood–brain barrier [43].

Our results demonstrate that *S. suis* EVs suppress the immune response and promote pathogen evasion through multiple mechanisms. In light of these observations, we hypothesized that the pure vesicle contents could produce similar effects, which led us to treat the EVs differently in subsequent experiments. Ultrasonically lysed extracellular vesicles (LEV), lacking an intact membrane structure, contained all active vesicle contents, while heat-inactivated extracellular vesicles (HEV), which lacked both an intact membrane and active vesicle contents, served as negative control. The results were unexpected, as LEV pre-incubation led to a significant increase in the transcriptional levels of cytokines and MHC-II molecules, showing a trend completely opposite to that of the intact EVs (Figure 4A,B). We speculate that the immunosuppressive components carried by EVs require an intact membrane structure to enter specific cellular compartments and exert their effects. In LEVs, membrane disruption may lead to the premature release of their contents, allowing immunogenic proteins to be recognized by pattern recognition receptors (PRRs) on the cell membrane, thereby triggering pro-inflammatory signaling pathways. However, immunosuppressive factors that originally depended on vesicle integrity for transport were unable to effectively reach their intracellular target sites due to the loss of membrane protection, leading to the failure of their inhibitory function and ultimately resulting in the observed experimental outcomes. As previous studies have shown [9,44], EVs exhibit dual functionality in host–pathogen interactions. On one hand, they can activate host immune defense mechanisms to eliminate pathogens, while on the other, they facilitate bacterial immune evasion strategies to establish persistent infections. These seemingly contradictory interactions maintain a delicate dynamic balance, with EVs serving as a central regulator that ultimately determines the infection outcome—whether it is acutely cleared or progresses into a chronic state.

In the previous study, we found that only extracellular vesicles (EVs) with an intact membrane structure can exert immune evasion. Therefore, we hypothesized that the immunosuppressive effects mediated by EVs may result from the internalization of specific substances secreted by *S. suis*, which are carried into macrophages by EVs with an intact membrane structure, leading to alterations in cellular metabolism. KEGG pathway analysis revealed (Figure 5D) that the differential metabolites of macrophages co-incubated with EVs, compared to the control group of normal cells, were predominantly concentrated in the glutamate metabolism, ammonia recycling, and citrate metabolism pathways. The clustered heatmap (Figure 5C) reveals a decrease in glutamine and glutathione levels within the glutamate metabolism pathway. Notably, glutamine plays a crucial role in macrophage activation and bacterial clearance. Glutamine is primarily synthesized from glutamic acid by the action of glutamine synthetase and is abundantly present in the liver, muscle, and other tissues and organs [31]. Glutamine may modulate macrophage clearance of pathogenic bacteria through various mechanisms. Ren et al. [45,46,47] reported that succinic acid produced during glutamine catabolism promotes M1 polarization of macrophages. M1-type macrophages primarily produce pro-inflammatory cytokines and reactive oxygen species, which enhance antigen presentation, thereby exerting bactericidal and anti-tumorigenic effects. Furthermore, glutamine also participates in the activation of key polarization signals, such as protein kinase B and AMP-activated protein kinase. These polarization factors regulate macrophage immune responses through multiple cellular pathways [48,49]. Glutamine plays a role in both glutamate metabolism and ammonia recycling pathways.

Additionally, another important metabolite in glutamate metabolism, glutathione, was significantly reduced following preincubation with EVs. Glutathione (GSH) is synthesized from glutamate, cysteine, and glycine in a two-step enzymatic process and acts as a key intracellular antioxidant [50]. Macrophages utilize reactive oxygen species (ROS) and reactive nitrogen intermediates (RNI) in their antimicrobial mechanisms. Superoxide, a form of ROS, can also damage macrophages during bacterial clearance; in such cases, intracellular GSH binds to excess ROS, protecting the host from oxidative damage. Nitric oxide (NO) is a key member of the RNI family and is widely recognized as an important bactericidal agent. However, its biological activity is short-lived, and it is rapidly degraded into nitrate and nitrite. Recent studies, however, have shown that NO can combine with GSH to form S-nitroso glutathione (GSno), which can rapidly decompose to produce NO and kill pathogenic bacteria upon macrophage encounter with the pathogen [51]. And besides killing pathogenic bacteria, macrophages are important antigen-presenting cells. Following the phagocytosis of pathogenic bacteria, macrophages hydrolyze them into antigen fragments within the endosomal/lysosomal system and form complexes with MHC-II molecules, activating CD4+ T cells and stimulating specific immunity. Furthermore, intracellular glutathione levels influence antigen delivery. When the intracellular level of GSH decreases, Ag processing is inhibited and the formation of MHC-II molecules is blocked, which in turn prevents antigen delivery [52]. Intracellular GSH levels also play a significant role in the formation of inflammatory factors. N-acetylcysteine (NAC), a precursor of GSH with notable anti-inflammatory activity, blocks nuclear factor-κB (NF-κB) activation by sequestering it in the cytoplasm via regulation of IκB. NF-κB is a transcription factor for inflammatory cytokines such as *IL-1, IL-6*, and *TNF-α* [53]. In the present study, we observed a significant decrease in glutathione and glutamine, suggesting that EVs may attenuate macrophage-mediated killing during subsequent *S. suis* infections via this pathway.

Another prominently altered metabolic pathway is the citric acid cycle, also referred to as the tricarboxylic acid cycle (TCA cycle). Since a variety of substrates such as amino acids and fatty acids can participate in the TCA cycle, they constitute the center of cellular metabolism and play an important role in the immune response [54]. Citrate is a pivotal intermediate metabolite in the TCA cycle, and several previous studies have demonstrated that truncation of the TCA cycle in activated dendritic cells (DC) and M1-type macrophages result in citrate accumulation [55]. This occurs due to the transcriptional inhibition of isocitrate dehydrogenase (Idh). Citrate generates reactive oxygen species (ROS) and nitric oxide (NO) during subsequent transformations, which play a role in bacterial sterilization [56]. Additionally, citrate is involved in the formation of prostaglandins (PG), which have been identified as key drivers of inflammation [57]. Citrate is subsequently converted to isocitrate, which is then decarboxylated to form α-ketoglutarate (α-KG). α-KG exerts an anti-inflammatory response in macrophages. Liu et al. [58] reported that α-KG inhibits IκB activation, which is required for the NF-κB pathway, thereby suppressing proinflammatory responses. A significant increase in isocitrate was observed in the present study, indicating that EVs co-incubation reduces macrophage-mediated bacterial killing by promoting the conversion of citrate to isocitrate.

Furthermore, certain metabolic pathways, such as arginine and proline metabolism, as well as the Warburg effect [59], were not significantly altered after EVs co-incubation with macrophages. These pathways are also involved in cellular immune responses but are not discussed in detail here. These results support our hypothesis that EV internalization into macrophages impairs antigen presentation and inflammatory factor production by modulating cellular metabolism, thereby attenuating the host immune response and promoting the establishment of a persistent bacterial infection.

## 5. Conclusions

In summary, this study demonstrated that EVs from *Streptococcus suis* (*S. suis*) contribute to enhanced bacterial pathogenicity. By disrupting macrophage metabolism, EVs suppress host innate immunity and promote bacterial survival. In parallel, they enhance the capacity of *S. suis* to colonize host cells. Moreover, results from animal infection models also supported the above conclusions. Collectively, these findings shed light on a previously unrecognized mechanism of *S. suis* pathogenicity and offer novel insights for the prevention and control of porcine streptococcal infections.

## Figures and Tables

**Table 1:** The primer sequences utilized in this study.

Primer Name	Primer Sequence
*H2-Ab1*-F	CAGTGACAGATTTCTACCCAG
*H2-Ab1*-R	GTCCAGTCCCCATTCCTAA
*H2-Aa*-F	GCAGACGGTGTTTATGAGA
*H2-Aa*-R	AGAAGGGATGAAGGTGAGA
*H2-Eb1*-F	CTTCTACCCTGGCAACATT
*H2-Eb1*-R	CCACTCTGAGGAACCGTCT
*H2-Ea*-F	TCTTGGGTTGTTTGTGGGT
*H2-Ea*-R	CTCCTTGTCGGCGTTCTAC
*TNF-α*-F	CTCTTCTGTCTACTGAACTTCGGG
*TNF-α*-R	GGTGGTTTGTGAGTGTGAGGGT
*IL-1β*-F	TGTGATGTTCCCATTAGAC
*IL-1β*-R	AATACCACTTGTTGGCTTA
*IL-10*-F	TGCTATGCTGCCTGCTCTTA
*IL-10*-R	GGCAACCCAAGTAACCCTTA
*IL-8*-F	TGTTGAGCATGAAAAGCCTCTAT
*IL-8*-R	AGGTCTCCCGAATTGGAAAGG
*iNOS*-F	CACCCAGAAGAGTTACAGC
*iNOS*-R	GGAGGGAAGGGAGAATAG
*Icam1*-F	GATGGCAGCCTCTTATGTT
*Icam1*-R	GCTTGTCCCTTGAGTTTTA
*Vcam1*-F	CGTCATTATCTCCTGCAC
*Vcam1*-R	GTGCCTGGCGGATGGTGTA
*GAPDH*-F	AGGTCGGTGTGAACGGATTTG
*GAPDH*-R	TGTAGACCATGTAGTTGAGGTCA

## Data Availability

The original contributions presented in this study are included in the article/Supplementary Material. Further inquiries can be directed to the corresponding authors.

## Supplementary Materials

The following supporting information can be downloaded at https://www.mdpi.com/article/10.3390/microorganisms13112469/s1, Figure S1: Relative mRNA expression levels of the MHC-II–related *H2* gene cluster and *ICAM-1* in RAW264.7 cells after 4 h of co-incubation with 50 μg/mL EVs.
