# Supplementary material for: Extracellular Vesicles from Streptococcus suis Promote Bacterial Pathogenicity by Disrupting Macrophage Metabolism
Source: Microorganisms. 2025 Oct 29;13(11):2469. doi: 10.3390/microorganisms13112469 (PMC12654174; doi:10.3390/microorganisms13112469)
Supplement: Supplementary file 1 [file microorganisms-13-02469-s001.zip › microorganisms-3862779-supplementary.pdf]

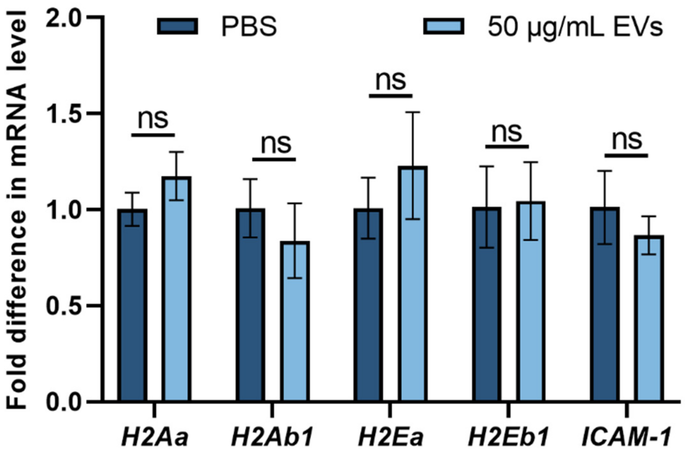

Figure S1. Relative mRNA expression levels of the MHC-II–related *H2* gene cluster and *ICAM-1* in RAW264.7 cells after 4 h of co-incubation with 50 μg/mL EVs. PBS-treated cells were used as the control group. Data are presented as the mean ± standard deviation (SD). Statistical significance: ns, $P > 0.05$, $^{*}P < 0.05$, $^{**}P < 0.01$, $^{***}P < 0.001$.
